# Barriers, facilitators, and solutions to familial hypercholesterolemia treatment

**DOI:** 10.1371/journal.pone.0244193

**Published:** 2020-12-23

**Authors:** Laney K. Jones, Amy C. Sturm, Terry L. Seaton, Christina Gregor, Samuel S. Gidding, Marc S. Williams, Alanna Kulchak Rahm

**Affiliations:** 1 Genomic Medicine Institute, Geisinger, Danville, PA, United States of America; 2 Center for Pharmacy Innovation and Outcomes, Geisinger, Danville, PA, United States of America; 3 University of Health Sciences and Pharmacy in St. Louis, Saint Louis, MO, United States of America; 4 Mercy Clinic—East Communities, Saint Louis, MO, United States of America; Tabriz University of Medical Sciences, IR Iran, ISLAMIC REPUBLIC OF IRAN

## Abstract

**Background:**

Familial hypercholesterolemia (FH) is an inherited lipid disorder that confers high risk for premature cardiovascular disease but remains undertreated. Causes are multifactorial and multilevel, ranging from underprescribing (at the clinician-level) to medication nonadherence (at the patient-level). We evaluated patient and clinician stakeholder barriers and facilitators for treatment of FH to explore possible solutions to the problem.

**Methods and results:**

Semi-structured interviews and focus groups guided by the Practical, Robust, Implementation and Sustainability Model (PRISM), were conducted with 33 patients and 17 clinician stakeholders across three healthcare systems. A total of14 patients and 9 clinician stakeholders participated in on-site focus groups and the remainder were individual interviews. Transcripts were coded using an iterative process to create a static codebook. We characterized patient and clinician stakeholder barriers into three categories: medical care-, medication-, and life-related. Feasibility of brainstormed solutions varied and was not always representative of the needs of all stakeholders. Patients suggested a need for childhood screening for FH and doctors being persistent about the importance of treating FH, creation of a patient peer group, data transparency, advocacy, and policy changes that would enable patients to receive better treatment. Clinician stakeholders suggested the need for clinical champions. Both groups of stakeholders discussed the need for education about FH.

**Conclusions:**

Proposed solutions to improve treatment of FH proffered by participants in this study included resources for both patients and clinician stakeholders that clarify cardiovascular disease risks from FH, develop programs to screen for and identify FH at younger ages, and foster open conversations between patients and clinicians about treatment.

## Introduction

Familial hypercholesterolemia (FH) is a genetic lipid disorder that confers high risk for premature atherosclerotic cardiovascular disease (ASCVD). Because of exposure to elevated lipid levels from birth, FH contributes to higher risk for ASCVD than other causes of dyslipidemia, at all levels of LDL-cholesterol [1]. Preventing ASCVD in individuals with FH usually requires lifelong adherence to lipid-lowering therapies [2]. When FH individuals receive treatment and have sustained lipid values at or below target levels, ASCVD risk is reduced to equivalence with ASCVD risk in the general population [3]. The 2018 AHA/ACC Cholesterol Guidelines recommend statins as first-line therapy for FH; however, statin therapy alone is often insufficient at achieving target lipid levels recommended to prevent ASCVD [2].

Unfortunately, most individuals do not receive optimal treatment for their FH. Data from the CASCADE FH Registry, a nationwide registry of United States lipid clinics, indicates that fewer than half of the 1,295 enrolled individuals were prescribed a high-intensity statin therapy (42%) or were receiving more than one lipid-lowering medication (45%) [4]. There are similar reports of undertreatment of FH in other countries (China, Netherlands, Greece) with an average of 48–64% receiving high-intensity therapy [5–7]. These data highlight the need to understand the reasons for suboptimal use of lipid-lowering medications in the FH population.

The reasons for insufficient use of statin therapy are multifactorial and multilevel, ranging from underprescribing (at the clinician-level) to medication nonadherence (at the patient-level). Patient and clinician stakeholder barriers and facilitators for hypercholesterolemia management across this continuum have been previously described [8–14]. General practitioners identified barriers for statin prescribing including concerns about out-of-pocket costs for patients, clinician workload (e.g., brief office visits and high patient complexity), patient refusal of treatment, and contradicting clinical practice guidelines [12]. A qualitative synthesis of patient barriers regarding treatment adherence identified six barriers [8]: ‘mismatch between perceived and actual risk’, ‘concerns about the use of lipid-lowering medication’, ‘prioritization of medication over lifestyle treatment’, ‘lifestyle treatment is difficult to comply with’, ‘prioritization of other life events’ and ‘inadequate and/or incorrect knowledge of treatment advice’ [8].

While these prior studies have identified barriers and facilitators to treatment, the populations studied had non-specific hyperlipidemia, and FH patients were not identified or analyzed separately to identify condition-specific issues. The FH population has not been specifically studied with regard to improving overall treatment (although some smaller studies have examined treatment adherence in FH). While some overlap with other lipid disorders could be anticipated, the higher risk for ASCVD, the need to use more intensive lipid-lowering therapies, and the inherited component of the disease, including the ability to make a diagnosis based on genetic testing, could lead to barriers and facilitators not previously identified. Prior studies also have not included stakeholders beyond patients- and clinician-level stakeholders; thus, missing opportunities to identify barriers and facilitators at the system level (i.e., hospital systems). Furthermore, previous studies have not used an implementation science framework to guide the analysis of barriers and facilitators. This project utilized the pragmatic, robust implementation and sustainability model framework (PRISM) to provide guidance across these multilevel contextual factors; such as, external context including resources and guidelines and internal context including patient and clinician characteristics and values [15]. The study goal was to identify potential solutions that address the multilevel barriers and build on facilitators to improve FH-specific treatment.

## Methods

A protocol of this study has been published previously [16]. A brief summary of the methods is included here. This study was approved by Geisinger, Mercy Health System, St. Louis College of Pharmacy (ceded to Geisinger), and Washington University in St. Louis (ceded to Geisinger) Institutional Review Boards. Participants provided verbal consent by agreeing to participate prior to the start of the interviews or focus groups. If participants agreed, the interview and focus group were conducted.

### Participants and recruitment strategy

Participants were recruited for this study form three sites: an integrated delivery system, a community-based health system, and an academic medical center.

Patient stakeholders with clinically diagnosed FH, based on a diagnosis code for FH in the medical record, were recruited from all three sites. At the integrated delivery system, individuals with a genetic diagnosis were recruited from the MyCode® Community Health Initiative that returns actionable genetic results to individuals, including those for FH [17]. Clinician stakeholders included individuals who were directly involved in the care of FH patients (cardiologists, endocrinologists, primary care physicians, mid-level clinicians, pharmacists, and genetic counselors). Clinician stakeholders were recruited via two methods: 1) they were clinicians of the eligible patients for this study, or 2) the study team emailed heads of departments at each site to ask for participation in the study (some department heads also volunteered to participate). System-level stakeholders were also targeted for this study (e.g., health plan representatives or system and administrative leads), but due to limited participation their results were analyzed with the clinician-level stakeholders. After completing the interview phase of the project, each stakeholder was invited to participate in follow-up, on-site focus groups at each location.

### Data collection

Semi-structured interviews were conducted by phone by one investigator (L.K.J.) using standardized interview guides (S1 and S2 Files). Guided by the PRISM framework, individualized interview guides were created for each stakeholder group to discover barriers and facilitators to treatment. Interviews were conducted until thematic saturation occurred in each stakeholder group [18]. Demographic data were collected during the interview.

One focus group per stakeholder group (patient or clinician stakeholders) was conducted at each site (S3 File). Only one system stakeholder volunteered to participate in a focus group and therefore participated in the clinician stakeholder group. The purposes of the focus groups were to elicit feedback from each group on barriers and facilitators identified through the interviews from all stakeholders and to discuss potential solutions to overcome these barriers and leverage identified facilitators.

### Data analysis

Interviews and focus groups were digitally recorded, transcribed verbatim, and uploaded into Atlas.ti (www.atlasti.com) for analysis. The first round of coding used an *a priori* codebook developed from the interview guides, stakeholder summaries, and PRISM constructs. If the pre-defined codes did not fully encompass emergent themes, then additional codes were created from the text using inductive analysis [19]. Study team members independently coded 2–3 transcripts and then discussed their coding, adjusted the codebook, and created a working analytic framework by grouping codes into categories and themes. This iterative coding process continued until the code list was static, all transcripts were coded, and the analytic framework was finalized.

## Results

### Demographics and experience with FH

**Fig 1:**
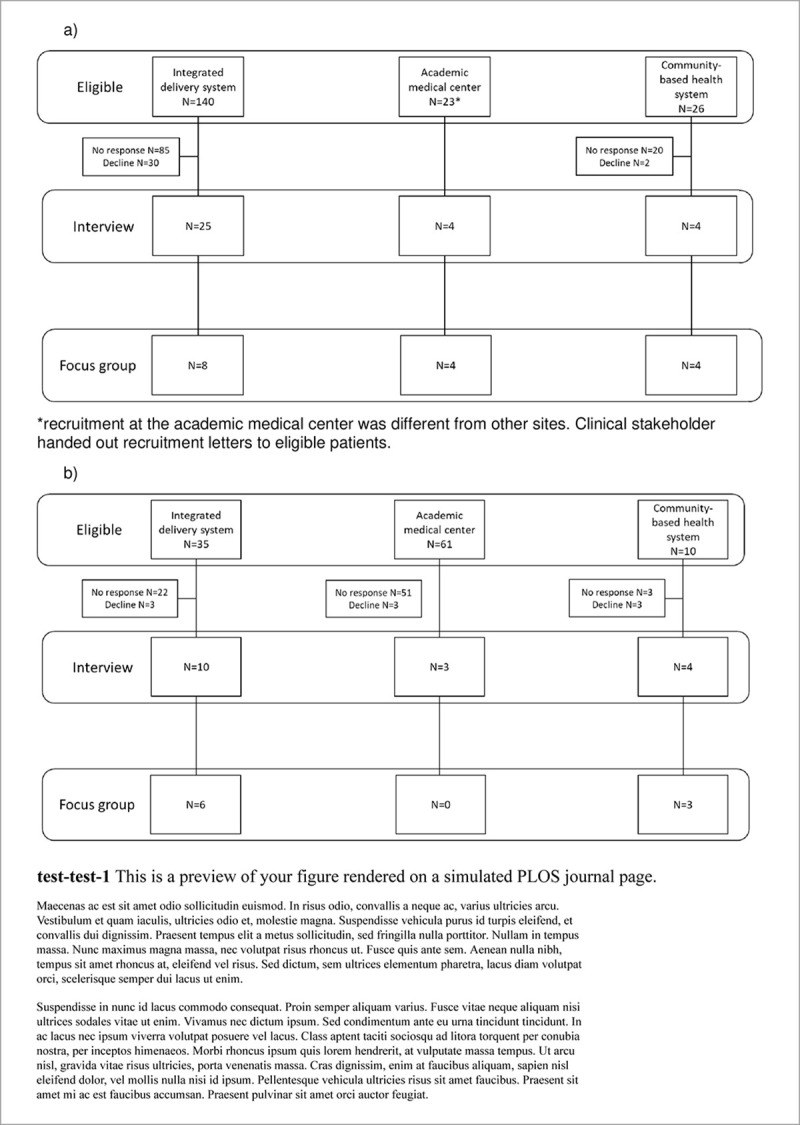
A) Patient stakeholder recruitment for interviews and focus groups B) Clinical stakeholder recruitment for interviews and focus groups. *Recruitment at the academic medical center was different from other sites. Clinical stakeholder handed out recruitment letters to eligible patients.

A total of 33 patients and 17 clinician stakeholders (5 system stakeholders; 4 were previous clinicians) participated in semi-structured interviews. Of those, 14 patients and 9 clinician stakeholders (1 system stakeholder) participated in subsequent on-site focus groups at their respective organizations (Fig 1).

Table 1 lists patient stakeholder demographics for the interviews and focus groups. Overall, patient participants tended to be women, older (age > 65 years), those with some college education, and individuals with diverse household incomes. About half had private insurance, and the other half public insurance, with a handful of uninsured participants. Patients described a delay in learning the specific name for their cholesterol diagnosis as FH, but discussed knowing about their high cholesterol earlier from their family history or routine bloodwork. One individual describes their family’s experience with this condition:

*“My grandfather was 60; my dad was 61; his brother*, *who is my uncle*, *was 53; his son*, *[who] was my cousin*, *was 43; and my other cousin was only 38*. *And they all died of massive heart attack*.*” (Integrated delivery system patient interview #10*)

**Table 1:** Demographics.

a) Patient stakeholder demographics				
Demographics			Interview (n = 33)	Focus group (n = 14)
Female			24 (73%)	10 (71%)
Age (years)				
	18 to 64		19 (58%)	6 (43%)
	65 or older		14 (42%)	8 (57%)
Education				
	College graduate		11 (33%)	7 (50%)
	High school graduate		3 (9%)	1 (7%)
	Post graduate education or graduate degree		7 (21%)	4 (29%)
	Some college		8 (24%)	2 (14%)
	Some high school		2 (6%)	
	Trade/technical/vocational school		1 (3%)	
	Trade/technical/vocational school & some college		1 (3%)	
Health insurance coverage				
	Federal or state insurance		17 (52%)	7 (50%)
	No insurance		1 (3%)	
	Private insurance		16 (48%)	7 (50%)
White race			32 (97%)	13 (93%)
b) Clinician stakeholder demographics				
Demographics			Interview (n = 17)	Focus group (n = 9)
Female			9 (53%)	3 (33%)
Type				
		Cardiologist	2 (12%)	2 (22%)
		PCP/Internal Medicine*	6 (35%)	3 (33%)
		Genetic Counselor	2 (12%)	1 (11%)
		Pharmacist*	5 (29%)	2 (22%)
		Health Plan	2 (12%)	1 (11%)
Average length in position, years (SD)			6.3 (9)	8.2 (9.4)
Average length at organization, years (sD)			7.6 (9.9)	10.2 (11.7)

*Two individuals in these categories are now directing these departments.

When asked about how they were diagnosed with FH, 15 stated they had a genetic diagnosis (12 through the MyCode Initiative [17]), 14 through clinical criteria, and 4 were not asked (these were the first individuals to be interviewed and this question was not originally part of the interview guide). One individual who had a clinical diagnosis said they had some genetic testing through a direct-to-consumer genetic testing company, but the study team was unable to discern if that test reported a positive result for FH. One individual who was not asked about genetic testing stated that a daughter had a positive genetic test result, but the individual did not disclose additional specifics to confirm testing type or that result was specific to FH. Almost all individuals (31/33) were receiving treatment with lipid-lowering therapy and 12 of 31 (39%) were treated with a PCSK9 monoclonal antibody. About two-thirds were being treated for primary prevention (22/33) and the remaining third for secondary prevention following a cardiovascular event. Of the 14 individuals who subsequently participated in the focus groups (subset of individual interview participants), based on self-report, half (7/14) were receiving PCSK9 monoclonal antibodies, 57% (8/14) reported side effects attributed to lipid-lowering medications, 64% (9/14) reported trouble accessing medications, and 64% (9/14) ever having an LDL-C below 100 mg/dL.

Clinician stakeholder demographics are also available in Table 1 for the interviews and focus groups. Overall, the clinician stakeholder participants interviewed were about 50% female with the most frequent participants being pharmacists and primary care physicians. They had an average of 6 years in their position, and an average of 7 years at their respective institutions. Of the 9 stakeholders who further participated in the focus groups (subset of individual interview participants), two-thirds (6/9) were male practitioners and these practitioners had a longer time in their respective positions (8.2 years on average) and length at their institution (10.2 years on average). Most clinician stakeholders knew about the clinical criteria to diagnose FH, even though some were in roles in which they were no longer treating patients. Not all clinician stakeholders ordered genetic testing as part of their practice and clinician stakeholders at the integrated care system more often ordered genetic testing. Most recognized that FH is an inherited condition that requires counseling for family members. Most clinician stakeholders who saw patients indicated they follow treatment guidelines for therapy decisions. One individual described a personal approach to treating individuals with FH:

*“It’s different because FH is sort of a lifetime problem and the problems associated with having high levels [of] cholesterol over many*, *many years results in increased frequency of these problems and severity of these problems so we’re more aggressive in the therapy*.*” (Integrated delivery system stakeholder interview #1*)

Of the 9 clinician stakeholders who also participated in the focus groups, all remember having had some education about FH, but only 7 (78%) reported caring for a patient with FH, and 3 (43%) had difficulty achieving recommended lipid levels in their patients with FH.

### Facilitators and barriers to receiving treatment for FH (patient stakeholders)

Each patient reported a unique care experience for FH. Almost all expressed experiencing both satisfaction and frustration with their care at some point (Table 2). Many described feeling frustrated with their cholesterol care until they were diagnosed with FH, and others noted that lack of family communication about health issues contributed to delays in getting the FH diagnosis. However, all were glad to know that they have the FH diagnosis.

**Table 2:** Stakeholder reporting of facilitators, barriers, and solutions related to FH care.

**Facilitators**		
	Patient-level	Clinician-level
	• Great medical team • Good understanding of FH • Useful resources for FH	• Good knowledge of available treatments • Good understanding of genetic results • Clear diagnostic criteria for FH
**Barriers**		
	Patient-level	Clinician-level
Medical care-related	• Changing guidelines • Experiencing care gaps • Nondisclosure of family history	• Lack of sufficient evidence to support some methods of identification or treatment • Lack of awareness of FH
Medication-related	• Lack of insurance coverage for treatments • Lack of awareness of other treatments • Reluctance to take other treatments • Side effects experienced	• Difficulty convincing patients to adhere to prescribed medications • Lack of awareness of other treatments
Life-related	• Competing family demands • Loss of job/health insurance • Competing personal demands (e.g., other illnesses)	• Busy clinics (e.g., high patient volumes) • Lack of a cohesive medical record
**Suggested solutions**		
	Patient-level	Clinician-level
	• Patient education about FH • Childhood screening for FH • Data availability to patient • Doctors’ persistence in treatment recommendations • Creation of a peer group • Increased awareness of FH • Health policy changes	• Clinician education about FH • Clinician champions

#### Facilitators

Many patients reflected that what was most valuable for their care was finding a great medical team who listened, worked with them, responded to suggestions, was proactive and passionate about their health, and gave them time/did not rush them. Many described the diagnosis itself as a facilitator to understanding their care needs, as evidenced by one patient’s experience, “*Well [the FH diagnosis] explains a lot*. *Considering I’m an active person*, *not overweight*, *I don’t eat ridiculous*. *So*, *it kind of puts it in to perspective*.*” (Integrated delivery system patient interview #10)*.

Patient barriers to receiving appropriate FH treatment and cardiovascular risk reduction were grouped into three common categories as identified by the study team: medical care-, medication-, and life-related barriers.

#### Medical care-related barriers

Patients described perceived gaps in care that they received in terms of never being counseled on an appropriate diet for managing cholesterol and confusion/frustration that the evidence for treating cholesterol has changed over time. For example, one patient said, *“…back then*, *you know*, *I’m 75*. *Back then you really didn’t hear about high cholesterol*. *Not like today*, *you know” (Integrated delivery system patient interview #6)*. Another patient felt that her symptoms were ignored. *“…when I was discharged with the stent*. *The doctor said to me*, *“don’t let anybody tell you you’re not having a problem if you have chest pain I don’t care how many times you go to emergency room*, *you keep going*, *you know until somebody pays attention to you*.*” I went to the ER a total of 5 times and every time I say I got this chest pain and they do an EKG and they say*, *“you’re fine*. *And they send me home*.*” (Integrated delivery system patient interview #3)*. In addition to these perceived gaps in their care, some also faced logistical issues in terms of difficulty scheduling appointments and limited access to a nearby specialist for their condition.

#### Medication-related barriers

Patients discussed medication-related barriers including problems accessing medications (e.g. prior authorization and cost) and problems taking their prescribed medications (e.g. side effects and nonadherence).

Many individuals discussed having to obtain a prior authorization for their prescribed lipid-lowering medication including statins and PCSK9 inhibitors. This process was often confusing and time-consuming for them: *“…Like someone like in my position with the amount of history behind me shouldn’t have to be jumping through hoops and being turned on*, *like prior authorization after*. *Ironically the one thing got me approved was the fact that I had the genetic testing and I guess*,… *I was automatically approved then*.*” (Integrated delivery system patient interview #10)*. Another patient described how they were required to try and fail to improve on other medications prior to approval of new and more expensive medications: *“Well*, *they just kept denying it and stuff they wanted me try*, *try this and try that*. *And the doctor said that I just can’t I just tolerate—I can’t take the statins*. *I’ve tried everything else they wanted me to do*. *And finally they would*, *you know*, *decided they were going to pay for it*, *so it just took awhile*… *While they refused for pay for it*, *you know*, *… I wasn’t taking anything*.*” (Integrated delivery system patient interview #12)*. Even if approved for a medication that the doctor wanted them to take, sometimes the cost still prevented them from taking the medication. A small group of individuals discussed also having no insurance for some period of time which also made it very hard to receive proper medical care and treatment due to the cost of healthcare and medications.

Nonadherence (most of these medications are taken daily), side effects, and contraindications, such as pregnancy, were also noted by patient participants as preventing them from receiving or taking the medication their doctor prescribed. One patient described side effects they experienced: *“I just can’t take [statin]*, *I think*, *that’s the devil’s drug*. *And that was probably one of worst side effects [e*.*g*., *muscle pain]*.*” (Integrated delivery system patient interview #21)*.

#### Life-related barriers

Almost all patients described some event that occurred in their life as affecting their ability to care for their FH. The reasons were varied and included both medical and non-medical reasons. For example, one individual stated that her spouse had died which caused her to lose health insurance and she could no longer afford treatment and, in addition, had become the sole caregiver for her family. While others described work-life balance or *“I’ve been going through a lot of medical issues” (Integrated delivery system patient interview #16)*. One individual described a feeling of being indestructible in his youth: *“35 was really when I started paying attention and getting on statin drugs so you know between the age of 25–30*, *31–34 I ignored it and just said*, *“it’s not going happen*, *I’ll deal with it when I’m in my 50s*. *And that was the frame of mind in my 20-30s*. *I was indestructible and I … play[ed] basketball*, *maintained a healthy weight*, *and … was not symptomatic*.*” (Integrated delivery system patient interview #15)*.

### Facilitators and barriers to treating individuals with FH (clinician stakeholders)

Clinician stakeholders reported differences related to training and experience in caring for individuals with FH. When asked about barriers and facilitators to caring for individuals with FH, clinician stakeholders primarily answered by posing solutions. Therefore, barriers and facilitators were extrapolated by the study team from these solutions and were assigned the same categories (medical care-, medication-, life-related) as described by patients (Table 2).

#### Facilitators

Clinician stakeholders familiar with FH described how the diagnosis itself facilitated patient care. Once the diagnosis of FH was known, they reported being more aggressive in their management of the patient and in encouraging patients to reach out to family members for testing and treatment.

#### Medical care-related barriers

Lack of awareness and knowledge about FH within the medical community was a key barrier. One clinician stakeholder stated, *“I think one of issues [is that] we don’t think of family hypercholesterolemia as a sort of separate entity [and] we probably should*.*” (Community-based health system stakeholder interview #13)*. Some clinician stakeholders expressed belief that there is not enough evidence to support the use of genetic testing as a possible method to identify individuals with FH. Clinician stakeholders described that even if they believe the evidence for genetic testing, the cost of the testing makes it unobtainable by their patients.

#### Medication-related barriers

Once patients are diagnosed, clinician stakeholders described that they often must convince patients to take the prescribed medication, with many wanting additional medication options. *“One of the problems with the statins are that a large percentage of patients develop muscle aches and joint pain*. *And they are really the only medications that have [been] shown to [be] effective in reducing heart disease and secondary events*. *[Statins] are a very important drug[s] [but if] somebody can’t take them because they are having muscle aches and joint pain*, *that’s a problem*. *We actually need more medications that will effectively treat the cholesterol that might not have that particular downside*.*” (Integrated delivery system stakeholder interview #2)*. However, even as new medications have been developed to treat FH (e.g., the PCSK9 inhibitors), many clinician stakeholders reported being unfamiliar or uncomfortable with prescribing the medications. One clinician stakeholder articulated this struggle: “*From a therapeutics perspective there is probably an even greater lack of familiarity or unawareness of PCSK9 so that leads to some degree of hesitance to consider using those medications even though it is probably something that particularly for pharmacists can be learned relatively quickly and at the end of the day it’s a cholesterol medication that just happens to be injectable*. *It’s one of those things where you don’t know what you don’t know and because of that there is probably a baseline sense of discomfort or at least apprehension about using those types of therapies*.*” (Integrated delivery system stakeholder interview #8)*.

Medication-related barriers were often expressed by clinician stakeholders as patient nonadherence to medications that they had prescribed. Discussion and solutions to medication nonadherence focused on the patient’s responsibility rather than something in the clinician stakeholders’ power to address.

#### Life-related barriers

Clinician stakeholders described barriers related to their clinical practice life in terms of high patient volumes that make it difficult to complete every task for each patient they see. In addition to this heavy workload, they also discussed how the lack of a cohesive medical record for an individual patient makes care difficult because they do not have all the information they need about the patient to make decisions. Some described that even when the information for the patient is available, the necessary tests have not been performed to allow them to make adequate decisions about the patient’s health (e.g., lipid panel).

*“I think I would just say that the demands made on people delivering the care make it exceedingly difficult to deliver good care and that if you’re going to ask a physician to see 20 people a day*, *do 5 treadmill tests*, *and read 7 echocardiograms*. *It’s really hard in the middle of all that to use evidence-based medicine to change therapy*, *discuss those changes with the patients*, *convince them that it is important*, *write the prescriptions*, *set up the follow-up labs*, *set up the follow-up visit*, *and move on*. *You know it’s just really tough with the volume of people that we’re all expected to see*.*” (Integrated delivery system stakeholder interview #1*)

One clinician stakeholder proffered the theory that limited attention is paid to FH by clinician stakeholders because spending time on prevention is a hard sell to their healthcare organization.

### Proposed solutions from identified facilitators and barriers

In the focus group, we presented the barriers and facilitators and asked participants about possible solutions and their feasibility. If solutions were not proposed, we suggested potential solutions that arose during the interviews to solicit reaction (Table 2). All participants who attended the focus groups also participated in the individual interviews (described above). Individuals with FH and clinician stakeholders were also asked to think of solutions for how they would fix the barriers or implement identified facilitators that they had described during their individual interviews. Often these solutions were just initial ideas and lacked detail on what the solution would look like or how it would or could be implemented into care. Not all proposed solutions met every patient need or were feasible or realistic for all participants.

#### Solutions to implement facilitators or address barriers (patient-level)

The proposed solutions discussed by patients as potentially being helpful for them focused mostly on addressing medical-related and life-related barriers, but they did describe a few solutions to improve the medication-related barriers.

*Education about FH*. Patients described a need for more information about FH before and after they were diagnosed. Patients thought that early detection and cholesterol screening needed to be a larger part of the healthcare discussion for all individuals so that FH could be identified earlier. One recommendation was to have an intake questionnaire about cholesterol and family history, similar to screening for other conditions, before you see your doctor: *“They do those little computer [questionnaires] for depression and anxiety…if your family [has] heart attack or how is your diet*…*” (Integrated delivery system patient focus group #1)*. Other recommendations were for increased use of the patient healthcare portals, employee and community health screenings, and other educational campaigns to increase awareness, communication, and provide reminders about screening for cholesterol. One patient stakeholder suggested, *“You can hire me*. *We’ll just go out and teach all these people about it*.*” (Integrated delivery system patient focus group #1)*.

Patients also requested information on how to communicate and educate their family members about FH. One patient described how she planned to inform her family and advocate for screening and treatment: *“We have a family reunion every year; it is really big*, *a couple hundred people usually go and we meet at a hotel*. *This year's it is in Alabama*, *and it’s [been] in Louisiana*. *And what I requested already was a time to speak with them and set up a table to educate them because I figured it out*, *nobody told me that my dad and his brother had it*.*” (Academic medical center patient focus group)*.

*Childhood screening for FH*. Many patients reported wishing they had known about this when their children were younger because it has been hard to convince their adult children to now go for testing. Some barriers that have arisen for their adult children have been the lack of health insurance to cover the cost; however, these patients felt that if their adult child had been screened in childhood, they would have been covered by their parents’ policy. Another solution proffered was to *“screen at birth when they do the heel stick*.*” (Integrated delivery system patient focus group #1)*.

*Data availability*. Patients requested to see the data (e.g., personal health LDL-C and risk data) to inform their decision about their care. Some individuals described *“need[ing] to have [the] facts” (Integrated delivery system patient focus group #2)* about what might happen to them if they do not do what the clinician stakeholder recommends. Another patient made their decision to go untreated regardless of the data and stated, *“I know I’m taking a risk*, *but it’s a calculated risk” (Integrated delivery system patient focus group #1)*.

*Doctors’ persistence*. A few patients requested more persistence from their clinician stakeholders and suggested reminders and nudges that might have made them take the condition seriously at an earlier timepoint. Patients suggested that hearing about the importance of FH from more clinician might convince them to take action earlier for FH. Another patient focused on persistence over time from healthcare professionals to alert them about the consequences of not taking action.

*“I mean persistence*, *I think as long as you guys as an organization just keep pushing us as patients*. *Heightened awareness around it*, *and no*, *it's not going to go away*, *and could cost you your life if you don't take some action*. *I think your persistence is all we can ask for*. *A selfish guy like me*, *I thought I was indestructible in my 20s*. *It did take me until my 30s*, *my next kind of step in life*, *and I realized I better get some prescriptions going*. *So*, *I think your persistence will help us persevere*, *hopefully sustain a longer life*.*” (Integrated delivery system patient focus group #2*).

*Creation of a peer group*. Patients discussed the importance and enjoyment of meeting other individuals with FH. During the focus group individuals often asked questions of each other and compared notes about care and medications. While only one group mentioned an online support group to be helpful, this natural group discussion indicates the creation of a peer group may also be well received as a possible solution. For example, one patient stated related to the focus group that, *“it was very interesting to hear other people's stories*. *I don't know anybody else who has this*.*” (Integrated delivery system patient focus group #2)*.

*Increased awareness*. Patients also discussed the need for increased awareness about FH such as creation of a patient advocacy organization and having an influential member of society to be the spokesperson in the media about this condition. One patient stated:

*“I think someone has to get to an influential senator or someone who maybe can go to bat for some of this or maybe even the media*, *maybe the media who wants to do all these stories*, *maybe the media needs to do a story on*, *you know*, *Medicare*, *all this stuff is so wonderful*, *but look at these people who are being denied the correct medicine for them because they're on a government program*. *Maybe the media has to do it*, *I don't know*, *but it's really kind of sad*.*” (Academic medical center patient focus group*).

*Policy changes*. Patients described how the inconsistency and changing policies related to medications, insurance coverage, and cholesterol management made receiving treatment for FH difficult. Two individuals shared their thoughts on the high cost and restrictions that insurance companies have put on a newer class of medications to treat FH, the PCSK9 monoclonal antibodies.

*“Getting the cost of some of this stuff down*, *I mean*, *it's astronomical and I think they have*, *what they call a Repatha card where you can get it for 5 dollars a month*, *but the clinker is that when they passed the Obamacare*, *they eliminated Crestor and Repatha and all this stuff that if you're on a government sponsored program they will not give you a Repatha card*, *so you're at the mercy of your insurance company or your Plan D*, *or whatever it is*, *and they haven't been approving it*, *so it makes it very hard for people to really get this drug*, *which really isn't fair*.*” (Academic medical center patient focus group*)

*“I think getting after insurance companies is a thing*. *Good Lord*, *somebody can't afford $14*,*000 a year for needed medicine*.*” (Integrated delivery system patient focus group #1*)

#### Solutions to implement facilitators or address barriers (clinician stakeholder-level)

*Education about FH*. Clinician stakeholders discussed the need for additional education to help identify and treat individuals with FH in order to assume care for them or a place to refer these individuals to be cared for by a more specialized clinician: *“you said [the] incidence is 1 in 250*, *we need to do better in identifying who all these patients are because I know*, *you know*, *I have about 1200 or 1300 patients right now*. *I don't think I have 6 people diagnosed with it*, *but it sounds like I should*, *and I'm not sure where I'm missing it from*.*” (Integrated delivery system stakeholder focus group)*. Clinician stakeholders also expressed uncertainty around prescribing new lipid-lowering treatments: *“I certainly haven't prescribed the injectable medicines*, *but I think if someone needs them*, *I usually consult Cardiology and discuss that*, *because I don't have the experience with them*. *It is difficult to deal with them*, *I [have] got enough to deal with honestly*. *So anyway*, *that is where I am at right now*.” *(Community-based health system stakeholder focus group)*. Additionally, stakeholders described that if this information could be distributed to them through an electronic health record tool, that it would prompt them to consider this diagnosis or management. Others described continuing education credit or board examination questions about FH as a solution would encourage clinicians to learn about the condition.

*Champions*. Clinician stakeholders discussed the importance of clinical champions to promote awareness within their healthcare system. One clinician stakeholder shared, *“[Clinician stakeholder] was instrumental…he is a cardiologist who is the head of that department and he was instrumental with that marketing campaign [for a specific test] because he was the one who brought it here*.*” (Community-based health system stakeholder focus group)*. Another felt that prevention within healthcare also needed further support: *“I would never*, *you know*, *turn down any assistance in*, *you know*, *publicizing or promoting more prevention*.*” (Integrated delivery system stakeholder focus group)*.

## Discussion

Our study provides a multilevel contextualized view of the barriers and facilitators to treatment of FH experienced by patients and clinician stakeholders. While previous reports have identified barriers and facilitators [8–14], we utilized a two-step process of interviews followed by focus groups, to learn from these barriers and facilitators what solutions might work for these individuals in their context to improve relevance and generalizability to other healthcare settings. We found that both patient and clinician stakeholders proposed solutions to educate themselves and others about FH and recognized the need for increased visibility and awareness (patients) and champions (clinician stakeholder) to help increase understanding of FH in general. Patients also identified other solutions such as the persistence of doctors, early screening and detection, and data availability and accessibility that would help them make informed decisions about their care. Often, patients suggested solutions they think would have helped them to better achieve their anticipated treatment goals set by their clinicians. Clinician stakeholders described many patient-level barriers (e.g., convincing patients to take and adhere to their medications) that hindered their ability to treat FH even when they prescribed appropriate therapy. Proposed solutions were societal, system, or multilevel but require additional brainstorming, development, and testing prior to implementation into practice. An important lesson learned is that patients and clinicians often identified solutions that were not within their control and would require change at the healthcare system- or policy-level. These solutions were not always feasible or realistic in different settings or may not be generalizable to all patients or clinicians.

An overarching request for more information about FH was expressed by all stakeholders. Results from these interviews suggest the need for local patient peer support groups to help patients process medical information related to FH and increased awareness about FH throughout health care systems. Also, clinicians pointed to the importance of identifying local champions to pioneer this work in respective healthcare settings. The solutions proposed by patients and clinicians across three healthcare systems represent possible interventions to improve treatment of FH. However, any solution must be adapted to local context for successful integration into care processes.

Many of the solutions discussed by our participants have reported in other projects to improve the uptake of statins in hypercholesterolemia except for the policy solution [20] and in primary prevention of cardiovascular disease [21]. A systematic review by the first author (L.K.J.) found that 258 solutions used across 86 hypercholesterolemia studies significantly reduced LDL-C, increased rates of statin prescribing, and improved adherence to statin medications [20]. The next step to advance this study will include mapping proposed solutions to known implementation strategies such as the Expert Recommendations for Implementing Change (ERIC) compilation [22], and defining these strategies using Proctor’s recommendations [23] so that these strategies could be generalizable and translatable between implementers. The ERIC compilation is one method to organize and define implementation strategies to facilitate utilization of proposed solutions across different healthcare settings. Proctor and colleagues provide guidance for the reporting of implementation strategies; such as, how to name it, define it, and specify it to further improve generalizability and facilitate broader utilization of solutions [23].

The literature regarding system- and policy-level barriers and facilitators is sparse for the treatment of FH. Many patients and clinician stakeholders in our study discussed system- and policy-level barriers and facilitators (e.g., insurance coverage for medications, amount and quality of evidence for FH diagnosis and treatment, and heavy workload at clinic sites for clinicians) for the treatment of FH but offered very few solutions to address barriers or implement facilitators at this level. Even though we recruited five system-level individuals (four had previously worked as clinicians seeing patients with FH) to participate there was not enough information to include any distinct information that would inform future solutions from a system-level perspective. Future work should focus on identification of system- and policy-level barriers and facilitators and combining them with patient- and clinician stakeholder-level barriers and facilitators to develop comprehensive solutions and implementation strategies to improve clinical care for FH.

The strengths of this study include the diverse settings patient and clinician stakeholder perspectives from an integrated health delivery system, academic health center, and a community medical center. A major limitation of this study is the lack of enough stakeholders to comment on system-level barriers and facilitators for treatment of FH. Not all patient and clinician stakeholder demographic groups are represented, particularly those traditionally underrepresented in research, which could result in missing facilitators or barriers of relevance to these groups. Also, we were unable to host a focus group at the academic medical center due to a variety of factors (e.g., participant was no longer at that institution, not available at the selected time when the host was in St. Louis).

## Conclusions

Proposed solutions to improve FH treatment by participants in this study included resources for both patients and clinician stakeholders that clarify cardiovascular disease risks from FH, develop programs to screen for and identify FH at younger ages, and foster open conversations between patients and clinicians about treatment. Thus, it is not only important to identify barriers and facilitators to caring for individuals with FH but just as important to discuss and develop solutions. Solutions discussed with patient and clinician stakeholders should be turned into actionable interventions to improve care of individuals with FH. Future work is needed to define and implement these strategies into care and address multilevel barriers and facilitators identified in ours and previous work.

## Supporting information

S1 File (PDF)

S2 File (PDF)

S3 File (PDF)
