# Supplementary material for: Barriers, facilitators, and solutions to familial hypercholesterolemia treatment
Source: PLoS One. 2020 Dec 23;15(12):e0244193. doi: 10.1371/journal.pone.0244193 (PMC7757879; doi:10.1371/journal.pone.0244193)
Supplement: S1 File — (PDF) [file pone.0244193.s001.pdf]

Patient Stakeholder Interview Guide

Hello, my name is_____________and I am from [institution]. Thank you for taking the time today to help us with our study.

You were contacted for this interview because you have familial hypercholesterolemia (FH). We want to hear about your experiences with your care for FH. In particular, things that made getting your care easier or harder. We hope to explore and design strategies that help to encourage treatment for individuals with FH. This interview will take no longer than one hour.

As a reminder, your participation in this study is voluntary. I will be recording the interview to make sure I don't miss anything. Everything you tell me today will be kept confidential. In future reports of this work, we may use any quotes that explain a point particularly well, but nothing we use will identify you personally in any way. You do not have to answer any questions you do not want to, and you can stop this interview at any time. When we finish the interview, I will ask you to complete a short demographic survey.

Do you have any questions before we get started? *[answer questions]*

Do I have your permission to record the interview? (if yes) I will start the recorder and interview now. *[start recording]*

Opening Question

1. Tell me about when you learned that you had familial hypercholesterolemia.
    a. When did you find out you had this condition?
    b. Who told you that you had this condition?
    c. Did you receive genetic testing?
    d. Are you aware of anyone else in your family with high cholesterol?
        i. If so, who?
        ii. How has this condition impacted your family?
    e. What does having FH mean to you?
2. Tell me about when you learned you had high cholesterol.
3. Tell me about the type of care you received before and after learning about your FH diagnosis?
    a. Did your medications change?
    b. Did your doctor recommend additional therapy?

   c. How well do you feel your condition is being treated?

4. Have you had any heart or cholesterol-related events related to your FH? …Like a heart attack or stroke? Or been diagnosed with cardiovascular disease?

Familial Hypercholesterolemia – barriers and facilitators to care

5. Tell me about any obstacles that you have encountered related to caring for your FH.

   a. Tell me about access to:
      i. Appointments?
      ii. Doctors?
      iii. Medications?

6. Tell me about how you get your FH care covered?

   a. Does your health insurance cover treatment for your FH? (including visits, meds, etc.) Or other services?

   b. Have you experienced any problems?

   c. Has your doctor suggested anything that you haven't been able to get covered?

   d. Have you had to pay out of pocket for services?

      i. How has that affected your treatment?

7. Tell me about any part of your care for your FH that you have enjoyed.

   a. Anything you wish other patients could experience.

8. How do you manage your FH in the face of other health concerns?

Brainstorm strategies to promote uptake of evidence-based care of FH

9. What would make things easier to care for your FH? [based on obstacles described above]

   a. What sources of information have you used to learn more about FH?

   b. How could they be used in the medical system?

   c. Does this help you overcome all the barriers you have experienced (if you've experienced any)? What is left?

10. Are there any other things that we can do to improve this process?

Patient Demographics Questionnaire **ID__**

1. What is your sex?
   - ☐ Male
   - ☐ Female

2. Which of the following best describes your age group?
   - ☐ 18 to 24
   - ☐ 25 to 34
   - ☐ 35 to 44
   - ☐ 45 to 54
   - ☐ 55 to 64
   - ☐ 65 or older

3. What is the highest level of education that you have completed?
   - ☐ Grade school/junior high
   - ☐ Some high school
   - ☐ High school graduate
   - ☐ Trade/technical/vocational school
   - ☐ Some college
   - ☐ College graduate
   - ☐ Post graduate work or graduate degree

4. What type(s) of health insurance coverage do you have? (**answer all that apply**)
   - ☐ Private insurance (for example, Geisinger Health Plan or Blue Cross Blue Shield)
   - ☐ Medicaid
   - ☐ Medicare
   - ☐ Tricare/military

5. With which race do you identify yourself? (select all that apply)
   - ☐ Black or African American
   - ☐ White
   - ☐ Asian
   - ☐ American Indian or Alaskan Native
   - ☐ Native Hawaiian or other Pacific Islander
   - ☐ Other (specify)_________________[race other]

6. Do you consider yourself to be of Hispanic or Latino origin?
   - ☐ Yes
   - ☐ No

7. Are you currently married or living with a partner?
   - ☐ Yes
   - ☐ No

8. Are you currently working for pay?
   - ☐ Yes
   - ☐ No

9. Please indicate <u>your total combined</u> yearly **household** income before taxes.
   - ☐ < $15,000
   - ☐ $15,000 - $30,000
   - ☐ $30,001 - $50,000
   - ☐ $50,001 - $75,000
   - ☐ $75,001 - $100,000
   - ☐ $100,001 - $150,000
   - ☐ $150,001 - $200,000
   - ☐ > $200,000

Thank you for taking the time to talk with me today. We would like to send you a gift for your time. Would you like me to send this to your email or home address?
- ☐ **Email address: (fill in address)**
- ☐ **Home: (confirm mailing address)**

Are you interested in being contacted again to participate in a focus group?
- ☐ **Yes**
- ☐ **No**

Thank you!
