# Supplementary material for: Barriers, facilitators, and solutions to familial hypercholesterolemia treatment
Source: PLoS One. 2020 Dec 23;15(12):e0244193. doi: 10.1371/journal.pone.0244193 (PMC7757879; doi:10.1371/journal.pone.0244193)
Supplement: S2 File — (PDF) [file pone.0244193.s002.pdf]

Organizational Stakeholder Interview Guide

Thank you for agreeing to take part in this study. As a reminder, your participation in this study is voluntary. If you decide not to be in the study, it will not affect your employment. This interview will take no longer than one hour.

We want to understand barriers and facilitators to the care of patients with familial hypercholesterolemia (FH). We hope to explore and design strategies that promote evidence-based treatment for individuals with FH. We are looking to you for your perspective based on your expertise in the care of patients with high cholesterol.

This conversation will be recorded and transcribed, however your responses will remain confidential. You may also choose to not answer any question or end the interview at any time.

Do you have any questions before we begin? *[answer questions]*

Do I have your permission to record the interview? (if yes) I will start the recorder and interview now. *[start the recorder]*

Opening question

1. Can you please tell me about your current position at [institution]?
    a. Position/title:
    b. How long in position (years)?
    c. How long at organization (years)?
    d. Training/degree:
    e. Sex

General questions

2. In your role, how do you impact the treatment of patients with FH?

    a. If you treat patients, go to question 3.

    b. If you do not treat patients, how does your job affect individuals with FH?
        i. Do you set standards for FH treatment or identification? How do you make those decisions?
        ii. Are you involved in FH care as a payor?
            - How do you develop standards for coverage?
            - What evidence do you use?

3. Tell me about how you care for your patients with FH.

    a. How do you identify them?
        i. Do you use a tool?
        ii. Do you use genetic testing?

b. How do you treat them?
    i. What guidelines do you base your decisions off of?

4. How is this different then how you treat hypercholesterolemia patients?

    a. Is the identification different?

    b. Is the treatment different?

5. How do you explain this condition, FH, to your patients?

    a. What risks does this condition put on your patients?

6. Tell me how FH affects a patient's health compared to high cholesterol. *[clinician only]* Or population health. *[non-clinician only]*

    a. How would you characterize the burden of FH on a patient?

7. What your perception of the quality of the evidence regarding the treatment of FH?

Brainstorm strategies to promote uptake of evidence-based care of FH

Knowing this information that FH patients do not always receive the care that they need. We, the study team, want to explore how the health system can put into place a plan to encourage treatment of FH.

8. What would make things easier for you to care for patients with FH?

    a. If payor, what information do you need to help overcome barriers to treating patients with FH?

9. What has made things harder for you to care for patients with FH?

    a. If payor, what information do you need to help overcome barriers to treating patients with FH?

10. What do you need to help you get patients diagnosed with FH on evidenced-based treatment?

    a. Or to reach target goal?

    b. What barriers and concerns exist? Or do you foresee?

    c. How would this work within your current workflow?

11. *When we talked to some patients with FH, they felt they were already being adequately treated for their "high cholesterol."* What do you think would help you as a doctor or health system make sure these patients get adequate treatment? Or get to goal?

    a. Who would do that? Is that for patients? Doctors? Systems?

    b. What financial implications do you foresee FH patients having that could impact if they receive evidence-based treatment? How about financial implications that would impact the healthcare system?

    c. Do you think FH patient's cultural background or other factors would have an impact on their acceptance of the receiving treatment?

Organizational Characteristics

12. What type of support do you think it would take to implement evidence-based treatment for all FH patients in your health system?

    a. Technology? Workflow? Additional providers?

    b. How easy or difficult do you think it will be?

        i. How long do you think it will take?

        ii. Who are the key stakeholders?

        iii. What, if any, approvals are needed?

13. What evidence/proof/information do you need to being using evidence based treatment for patients with FH?

        i. What do you think others will need?

Thank you for taking the time to talk with me today. We would like to send you a gift for your time. Would you like me to send this to your email or home address?

- ☐ **Email address: (fill in address)**
- ☐ **Home: (confirm mailing address)**

Are you interested in being contacted again to participate in a focus group?

- ☐ **Yes**
- ☐ **No**

Thank you!
