# Supplementary material for: Barriers, facilitators, and solutions to familial hypercholesterolemia treatment
Source: PLoS One. 2020 Dec 23;15(12):e0244193. doi: 10.1371/journal.pone.0244193 (PMC7757879; doi:10.1371/journal.pone.0244193)
Supplement: S3 File — (PDF) [file pone.0244193.s003.pdf]

Familial Hypercholesterolemia Treatment Focus Group

[Welcome participants as they arrive. Have everyone fill out name tent and grab snacks/dinner as they arrive]

Welcome **(2 minutes)**

[Patients] Thank you all for coming today. We invited you because you have familial hypercholesterolemia and we are interested in hearing your thoughts on how we can improve care for you and other individuals with this condition. In previous interviews, we've heard about challenges patients with FH experience in trying to get care. These are opportunities for us to improve. Your thoughts, opinions, and ideas will help us to understand what we should be doing from your perspective.

[Stakeholders] Thank you all for coming today. We invited you because you are involved in the care of individuals with familial hypercholesterolemia. In previous interviews we've heard from patients and from representatives of groups like yours about barriers and challenges to deliver and receive care for FH. These represent opportunities to improve care. We are interested in hearing your thoughts on how to improve our processes for caring for individuals with this condition.

Consent/ground rules **(3 minutes)**

- Staff introductions & recording
- Consent elements
- Ground rules
    - All opinions matter, please try not to talk over each other
    - May call on individuals to make sure everyone has a chance to speak
    - May cut off a conversation to move on, if necessary
    - Be respectful - Disagree with ideas, not with people
    - Vegas rules - Do not discuss other participants' information or stories outside of the group
    - We are recording this discussion, but no names will be connected with what you say
- Plan for the day
    - Introductions
    - Go through some information about FH and treatments
    - Discussion of individual issues to understand your thoughts and ideas

Round table introduction of participants **(5 minutes)**

Let's go around the table. Please introduce yourself using your first name and tell the group what motivated you to participate today.

FH Overview **(5 minutes)**

[Show the 3 FH Information slide]

- FH is a common disorder
- It is an inherited disorder that is associated with a very high risk of early onset heart disease
- Many individuals are unaware of their condition

- Early and aggressive treatment is important to prevent disease
- Not everyone in the family will have very high cholesterol levels

- Even in our own system, we found that our patients are at risk
    - We found only half were prescribed high doses of statins and only a quarter met their target treatment goals

Discuss

- Thoughts?

Barriers and facilitators **(10 minutes)**

[Show Patient Problem Slide]

From interviews we have conducted and the literature, individuals have reported the following difficulties caring for their FH… We have listed a few examples on the screen.

Are we missing anything?

[Stakeholders] [Show Stakeholder Problem Slide]

From interviews we have conducted and the literature, individuals have reported the following difficulties caring for their FH… We have listed a few examples on the screen.

Are we missing anything?

Discuss

- Thoughts?

Questions **(10 minutes)**

[Show Raise Hand Slide]

Raise your hand…

[Patients]

- If you are currently receiving treatment for your FH?
  - If so and you feel comfortable can you tell us what you are taking?
- Who has tried treatment X?
  - Statins
  - Ezetimibe
  - PCSK9 inhibitors
  - Other medications
- Has anyone had side effects from these medications?
- Has anyone had trouble accessing these medications?
- How many individuals have had their 'bad' cholesterol below 100 mg/dL?
  - Current?
  - Ever?

[Stakeholders]

- If you have had a formal education on FH?
- If you have cared for individuals with FH?
- If you have had difficulty getting your patients to take medications prescribed for their FH?
- If you have had difficulty getting their patients to goal?

Defining implementation strategies **(45 minutes)**

[Show What can we do to help? Slide]

What can we do help with this problem?

[Patients] We know that there are problems that can make it difficult for you to take your medicines every day. How can we help?

[Stakeholders] From your perspective what changes could we consider that would make it easier for you to follow guidelines?

STRATEGIES (WRITE DOWN)

-

-

-

STRATEGY IDENTIFIED: probe for…

- *Actor*:
  - Who carries out the strategy?
- *Action*:
  - What actions, steps, or processes need to be put in place?
  - How do they get put into place?
- *Targets of action:*
  - Who is affected by the action?
  - How do we measure that action?
- *Temporality:*
  - When should the action be enacted?
  - What format should be it delivered?
- *Dose:*
  - How often should it be acted upon?
- *Implementation outcome affected:*
  - How can we measure it?
- *Justification:* we will collect afterwards…

PROMPTS (GO OVER IMPLEMENTATION STRATEGY FROM LITERATURE)

- If no answers, show an implementation strategy that affects patients.

**STRATEGY**					
ACTOR					
ACTION					
ACTION TARGET					
TEMPORALITY					
DOSE					
IMPLEMENTATION STRATEGY AFFECTED					

Closing **(10 minutes)**

Summarize discussion

Timeline

Task	Time (minutes)
Welcome	2
Consent/ground rules	5
Introductions	10
FH Overview	15
Barriers/Facilitators	25
FH Guidelines	35
Strategies	80
Closing	90
